# regSNPs-ASB: A Computational Framework for Identifying Allele-Specific Transcription Factor Binding From ATAC-seq Data

**DOI:** 10.3389/fbioe.2020.00886

**Published:** 2020-07-29

**Authors:** Siwen Xu, Weixing Feng, Zixiao Lu, Christina Y. Yu, Wei Shao, Harikrishna Nakshatri, Jill L. Reiter, Hongyu Gao, Xiaona Chu, Yue Wang, Yunlong Liu

**Affiliations:** ^1^Institute of Intelligent System and Bioinformatics, College of Automation, Harbin Engineering University, Harbin, China; ^2^Center for Computational Biology and Bioinformatics, Indiana University School of Medicine, Indianapolis, IN, United States; ^3^Regenstrief Institute, Indiana University School of Medicine, Indianapolis, IN, United States; ^4^Department of Medicine, Indiana University School of Medicine, Indianapolis, IN, United States; ^5^Department of Biomedical Informatics, The Ohio State University, Columbus, OH, United States; ^6^Department of Medical and Molecular Genetics, Indiana University School of Medicine, Indianapolis, IN, United States

**Keywords:** expression quantitative trait loci, allele-specific binding, transcription factor, ATAC-seq, functional single-nucleotide polymorphisms, computational biology, bioinformatics, transcriptional regulation

## Abstract

Expression quantitative trait loci (eQTL) analysis is useful for identifying genetic variants correlated with gene expression, however, it cannot distinguish between causal and nearby non-functional variants. Because the majority of disease-associated SNPs are located in regulatory regions, they can impact allele-specific binding (ASB) of transcription factors and result in differential expression of the target gene alleles. In this study, our aim was to identify functional single-nucleotide polymorphisms (SNPs) that alter transcriptional regulation and thus, potentially impact cellular function. Here, we present regSNPs-ASB, a generalized linear model-based approach to identify regulatory SNPs that are located in transcription factor binding sites. The input for this model includes ATAC-seq (assay for transposase-accessible chromatin with high-throughput sequencing) raw read counts from heterozygous loci, where differential transposase-cleavage patterns between two alleles indicate preferential transcription factor binding to one of the alleles. Using regSNPs-ASB, we identified 53 regulatory SNPs in human MCF-7 breast cancer cells and 125 regulatory SNPs in human mesenchymal stem cells (MSC). By integrating the regSNPs-ASB output with RNA-seq experimental data and publicly available chromatin interaction data from MCF-7 cells, we found that these 53 regulatory SNPs were associated with 74 potential target genes and that 32 (43%) of these genes showed significant allele-specific expression. By comparing all of the MCF-7 and MSC regulatory SNPs to the eQTLs in the Genome-Tissue Expression (GTEx) Project database, we found that 30% (16/53) of the regulatory SNPs in MCF-7 and 43% (52/122) of the regulatory SNPs in MSC were also in eQTL regions. The enrichment of regulatory SNPs in eQTLs indicated that many of them are likely responsible for allelic differences in gene expression (chi-square test, *p*-value < 0.01). In summary, we conclude that regSNPs-ASB is a useful tool for identifying causal variants from ATAC-seq data. This new computational tool will enable efficient prioritization of genetic variants identified as eQTL for further studies to validate their causal regulatory function. Ultimately, identifying causal genetic variants will further our understanding of the underlying molecular mechanisms of disease and the eventual development of potential therapeutic targets.

## Introduction

Expression quantitative trait loci (eQTL) analysis has developed over the years into a powerful tool to investigate the effects of genetic variants on gene regulatory networks, identify quantitative traits for complex diseases, and derive causal inference frameworks for genomic markers and gene expression (Fagny et al., 2017; van der Wijst et al., 2020). Specifically, eQTL analysis is designed to investigate how single-nucleotide polymorphisms (SNPs) in regulatory elements directly modify the abundance of a gene transcript. The major goal of an eQTL study is to reduce the large number of variants identified from a genome-wide association study (GWAS) to a list of potential causal SNPs for further investigation into how the locus contributes to disease. The validity of eQTL analysis has been demonstrated in multiple tissue types, in which high heritability has been observed in a large variety of gene transcripts (Nica and Dermitzakis, 2013).

Despite recent technological and methodological advances, eQTL analysis cannot distinguish between causal and non-functional variants that are in strong linkage disequilibrium (LD). In this study, we aim to identify functional SNPs in key regulatory regions that alter transcriptional regulation and thus, potentially impact cellular function. Such variants would be important for investigating the etiology of the associated disease and for identifying potential therapeutic targets.

Surveys of GWAS indicate that about 93% of disease- and trait-associated variants lie within non-coding sequences, especially in intergenic and intronic areas (Maurano et al., 2012). Notably, 76.5% of all non-coding GWAS SNPs are either within or in perfect LD with DNase I hypersensitive sites, which correspond to open chromatin regions that contain transcription factor (TF) binding motifs (Maurano et al., 2012). Such variants are likely to disturb gene expression by modulating transcriptional regulatory elements, including promoters, silencers, and enhancers (Cookson et al., 2009; Musunuru et al., 2010; Degner et al., 2012). Intronic variants can affect gene splicing by altering canonical splice sites, activating non-canonical splice sites, or changing splicing regulatory elements. In addition, intronic variants can also affect transcription regulatory motifs resulting in altered gene expression (Vaz-Drago et al., 2017). We previously developed a computational framework called regSNPs-intron that showed high accuracy in predicting disease-causing intronic SNPs (Lin et al., 2019). Similar methods are needed to prioritize intragenic variants that alter binding sites of key DNA binding proteins, such as TFs, as an efficient way of identifying candidate disease-causing SNPs.

Assay for transposase-accessible chromatin with high-throughput sequencing (ATAC-seq) is a high-throughput technology that employs an engineered Tn5 transposase to map genome-wide chromatin accessibility and nucleosome positioning (Buenrostro et al., 2013). In open chromatin regions, DNA-binding proteins protect DNA from DNase I enzyme digestion in DNase-seq or Tn5 transposase insertion (Thurman et al., 2012; Sung et al., 2016). Such protection often results in an altered pattern of enzymatic cleavage at the binding site compared to the flanking genomic region, which is referred to as a footprint. To date, many studies have inferred TF binding sites from ATAC-seq data by analyzing the digital genomic footprints left by DNA-binding proteins (Cavalli et al., 2016; D’Antonio et al., 2017; Wei et al., 2018). For example, the algorithms HINT-ATAC, DeFCoM, and Mocap each focus on identifying TF binding sites from sequencing-based footprint data (Chen et al., 2017; Quach and Furey, 2017; Li et al., 2019). While these existing methods predict TF binding events at the single base-pair resolution, none of these tools were designed to evaluate the impact of genetic variants on TF footprints. Furthermore, like DNase I, Tn5 transposase is reported to have specific sequence bias (Adey et al., 2010; Lu et al., 2017), although the impact of this bias on ATAC-seq footprinting profiles has not been systematically investigated.

Recently, the Sasquatch algorithm was developed to predict the effect of non-coding variants on TF binding by analyzing differences in DNase footprints between samples with different genotypes (Schwessinger et al., 2017). This method improved the statistical power by effectively eliminating biases resulting from variations in the footprint patterns of different transcription factors. Here, we propose to further analyze the differences in the ATAC-seq read distribution between two heterozygous alleles from the same individual. This strategy further reduces the biases of the sequencing variation on different experiments and sample types.

In our analysis, we first systematically screened for functional variants by applying a generalized linear model (GLM) based on the ATAC-seq data from two different cell lines. We further evaluated our findings using RNA-seq data from the same cell line, as well as publicly available chromatin interaction data (Teng et al., 2015). The overall strategy presented in this study provides evidence for functional SNP activity, which can serve as the basis for generating testable hypotheses for experimental validation. Ultimately, findings generated from regSNPs-ASB are expected to aid in understanding the molecular mechanisms of complex diseases.

## Results

**FIGURE 1:**
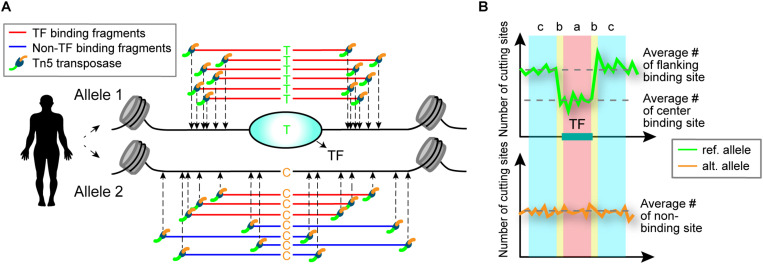
Schematic illustrating detection of allele-specific TF binding by ATAC-seq. **(A)** Schematic of allele-specific TF binding detected by ATAC-seq. Alleles 1 and 2 contain a single nucleotide difference in the TF-binding site, which results in preferential TF binding to allele 1. TF binding to the site prevents the Tn5 transposase from cleaving the DNA at that location. **(B)** The difference in TF binding is detected in ATAC-seq data by a characteristic footprint, observed as a dip in read counts at the binding site. An allele-specific binding (ASB) event is identified by the occurrence of this footprint in one allele (top) and uniform read counts in the other allele (bottom). The ASB regions are defined by transposase cut sites shown above the plot: a = TF-binding site, b = site shoulders, c = flanking region. The number of cut sites in region b are not considered in the regSNPs-ASB model to allow for variability in the size of the TF binding motifs.

Sequence variants within a TF-binding site may alter TF-binding affinity at that locus (Johnston et al., 2019). When functional SNPs are heterozygous and TFs preferentially bind to one allele, differential ATAC-seq cleavage patterns between the two alleles are expected to result (Figure 1). Based on this premise, we developed a computational model called regSNPs-ASB for detecting allele-specific differences in TF occupancy on a genome-wide scale.

### Overall Strategy and Data Preprocessing for regSNPs-ASB

**FIGURE 2:**
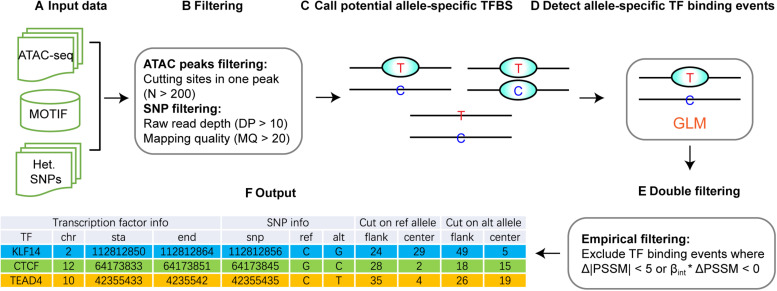
Workflow of regSNPs-ASB. **(A)** The input data for regSNPs-ASB are: (1) ATAC-seq raw read counts in a standard BAM format, (2) prior knowledge of TF binding motifs from the public domain, and (3) a vcf file with heterozygous SNP loci. **(B)** The first round of filtering considers only open-chromatin regions with a high amount of transposase cut sites and removal of SNPs with low mapping quality and read depth. **(C)** Call potential allele-specific TF-binding sites containing heterozygous SNPs. **(D)** Detect ASB events using GLM to identify allele-specific TF binding. **(E)** The second round of filtering requires the predicted ASB to have a high magnitude difference between reference and alternative alleles, as well as consistency in beta and PSSM values. **(F)** The output of regSNPs-ASB is a list of ASB events with associated TF information, regulatory SNP information, and corresponding ATAC-seq read counts from different alleles for each technical replicate.

The overall strategy of regSNPs-ASB is shown in Figure 2, which consists of six steps. First, open chromatin regions and SNPs were extracted from ATAC-seq data. Second, open chromatin regions and SNPs were filtered by the quality control and the total number of cutting sites in each open chromatin region, respectively. Third, potential allele-specific TF binding sites were extracted by merging the loci of heterozygous variants and TF binding sites. Fourth, significant allele-specific TF binding events were detected using GLM. Fifth, empirical filtering was performed to avoid false positive events. The final output is a list of ASB events with associated TF and regulatory SNP information.

To identify potential allele-specific transcription factor binding sites, we first conducted ATAC-seq experiments on patient-derived mesenchymal stem cells (MSC) and a breast cancer cell line (MCF7). Using the MACS2 algorithm (Feng et al., 2011), we identified 33,919 and 8,746 open chromatin regions in MSC and MCF7 cells, respectively. Within these regions, heterozygous SNPs were derived from the ATAC-seq BAM files using samtools (Li et al., 2009). After quality control filtering, 1,119,374 and 815,852 heterozygous variants were identified in the open chromatin regions of MSC and MCF7 cells, respectively (Table 1). In each open chromatin region, we used FIMO (Grant et al., 2011) to scan both reference and alternative sequences for putative transcription factor binding sites. In total, 3,874,515 and 1,725,376 candidate binding sites were identified from MSC and MCF7 cell lines, respectively. Among these, 40,475 binding sites contained heterozygous variants in MSC and 12,402 in MCF7.

**TABLE 1:** regSNP-ASB filtering summary.

Dataset	Chromatin accessibility regions	Heterozygous SNPs	Potential TF binding sites	Candidate allele-specific TF binding sites	Significant allele-specific TF binding sites	Filtered allele-specific TF binding sites	Regulatory SNPs
MSC	33,919	1,119,374	3,874,515	40,475	2417	406	125
MCF-7	8,746	815,852	1,725,376	12,402	922	122	53

### Detection of Allele-Specific TF Binding Events in MSC and MCF7 Cells

For each potential TF binding site containing heterozygous variants, we asked whether there were allele-specific differences in the open chromatin regions. To address this question, a generalized linear model (see section “Materials and Methods”) was implemented to examine the differences in the ratio of the cut site frequency in the flanking regions relative to the TF footprint (i.e., the flanks-to-footprint ratio) between the reference and alternative alleles (Figure 1B).

A negative binomial distribution was used in our model to account for the over-dispersion of the read counts. The coefficient of the interaction between the allele type (reference or alternative) and the region (flanking or footprint), β_int_, was used to evaluate whether the footprints on the putative TF-binding sites were significantly different between the two alleles. This analysis resulted in 2,417 candidate allele-specific TF binding sites in MSC and 922 sites in MCF-7 cells (FDR < 0.05). Using the position weight matrices (PWM) of the candidate TFs retrieved from the JASPAR database (Khan et al., 2018), we further calculated the position-specific scoring matrices (PSSM) scores (see section “Materials and Methods”) of the candidate allele-specific TF-binding sites for both reference and alternative alleles. Overall, we detected 406 and 122 candidate TF-binding sites in MSC and MCF7 cell lines with a significant β_int_ value and a sizable PSSM score difference (| deltaPSSM| > 5). Since each variant can potentially disrupt the binding sites of multiple TFs, 125 and 53 heterozygous SNPs in MSC and MCF7 were identified with the potential to disrupt TF binding. The number of TFs whose binding sites were disrupted by each heterozygous variant are shown in Supplementary Figure S1. A complete list of these variants can be found in Supplementary Tables S1, S2. In addition, the β_int_ values, the ATAC-seq read distribution on and flanking the putative sites, and the deltaPSSM, can be found in an RShiny website^1^.

**FIGURE 3:**
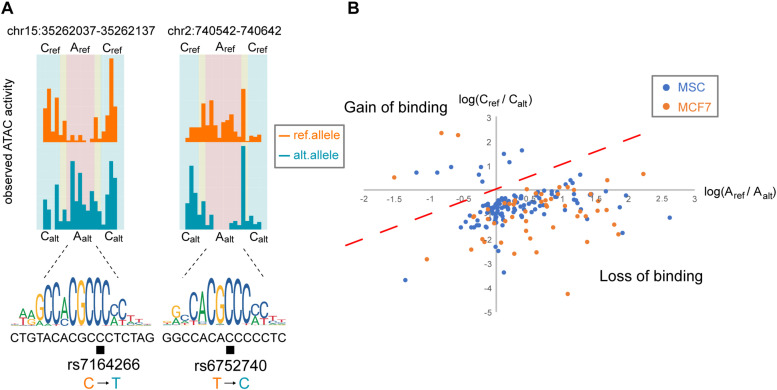
Allele-specific TF binding events occur throughout the genome. **(A)** Two examples of allele-specific TF-binding events in MSC are depicted by orange and blue histograms representing the observed ATAC-seq profiles for the reference and alternative alleles, respectively. The corresponding TF-binding motif is shown below the histograms, where the black box indicates the SNP locus. The left panel illustrates TF binding on the reference allele and the right panel illustrates TF binding on the alternative allele. Based on the regions defined in Figure 1B, we summed the corresponding read counts: A_ref_ = the number of reference allele reads binding at the TF-binding site; A_alt_ = the number of alternative allele reads binding at the TF-binding site; C_ref_ = the number of reference allele reads binding at the flanking region; C_alt_ = the number of alternative allele reads binding at the flanking region. **(B)** Log-ratio plot of A_ref_/A_alt_ vs. C_ref_/C_alt_. Points located in the lower right quadrant of the plot represent variants that result in a loss in TF binding, similar to the SNP shown in the right panel in **(A)**. Points in the upper left quadrant represent a gain in TF binding, similar to the SNP in the left panel in **(A)**.

Examples of the ATAC-seq signal distribution for two TF binding sites on the reference and alternative alleles identified by regSNPs-ASB are presented in Figure 3A. For SNP rs7164266, regSNPs-ASB detected a decrease in the footprint on the alternative allele. The matching score differences (deltaPSSM = 7.24) further supported the regSNPs-ASB prediction. Since rs7164266 occurs in a CTCF-binding motif, this finding suggests that the variant allele could potentially repress CTCF binding to this site. On the contrary, for rs6752740, a SNP located in a putative KLF14 binding site, a clear footprint was observed on the alternative allele, but not on the reference allele. This finding indicates that the rs6752740 SNP could enhance KLF14 binding, which was supported by the matching score differences (deltaPSSM = −5.48). The ratio of the number of ATAC-seq cutting sites between the two alleles on putative TF binding sites (X) versus the flanking regions (Y) was plotted for all of the identified events (Figure 3B). Taken together, these results indicate that the regSNPs-ASB algorithm can identify genome-wide allele-specific TF binding events from ATAC-seq data.

### Correlation of Allele-Specific TF Binding and Allele-Specific Gene Expression

We next asked whether the allele-specific TF binding predicted by regSNPs-ASB correlated with allele-specific gene expression. To address this question, we first identified likely target genes for the heterozygous variants that exhibited allele-specific TF binding. For putative promoter variants, the closest gene to each variant was considered as the target gene. However, because enhancer variants can be far away from their target genes, Chromosome Conformation Capture (3C) and its derivative techniques have become the major biochemical approaches to study such distal transcriptional regulation and chromatin interactions. Therefore, potential enhancer regulatory SNPs were combined with 3C experimental interaction data obtained from the 4Dgenome database. For this analysis, we focused on MCF-7 cells since 3C data was not available for MSCs. In MCF7 cells, 8 of the 53 heterozygous variants that exhibited allele-specific TF binding were located in the promoter or 5′UTR regions, while the other 45 SNPs were located in intronic or distal intergenic regions and were considered potential enhancer regulatory SNPs. For these 45 putative enhancer SNPs, 66 potential target genes were identified in the 4Dgenome database.

Allele-specific expression analysis for the target genes was conducted using heterozygous variants in the open reading frame of the putative target genes. We found that 75% (6/8) of the target genes associated with promoter variants and 39% (26/66) of the target genes associated with putative enhancer variants showed significant allele-specific expression (*p*-value < 0.01, Supplementary Table S7).

**FIGURE 4:**
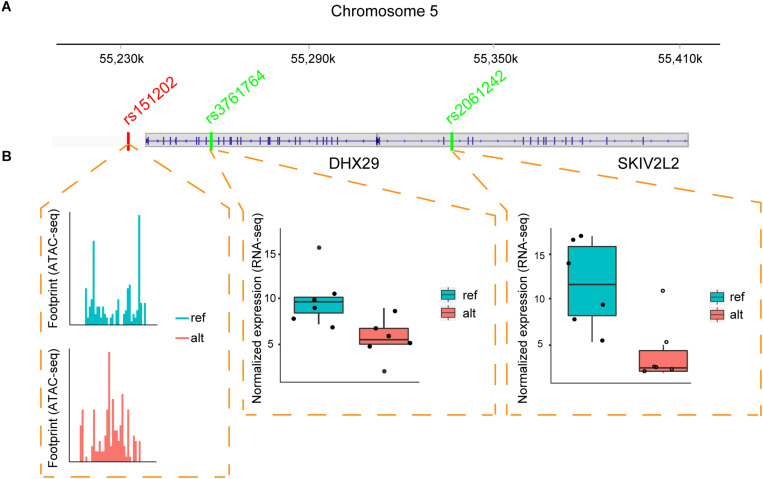
Regulatory SNP affects the expression of two target genes. **(A)** Illustration of the relative positions of the candidate regulatory SNP (red) and coding-region SNPs (green) located within two target genes (DHX29, SKIV2L2) in MCF-7 cells. Vertical blue lines indicate exons. **(B)** ATAC-seq and RNA-seq data suggest the regulatory SNP can drive an ASB event. The ATAC-seq footprint shows preferential TF binding on the reference allele at the regulatory SNP. Boxplots from RNA-seq data across six samples show the gene expression values of the two target genes is increased in the alternative allele.

One example of the chromatin interaction analysis is shown in Figure 4, where allele-specific binding on rs151202 was observed in the ATAC-seq data (Figure 4B). Motif analysis indicated that this SNP weakened the binding of transcription factor AP-2 gamma (TFAP2C). A previous 3C study reported that this SNP is located in a regulatory region that interacts with two target genes, *DHX29* and *SKIV2L2* (Teng et al., 2015). Interestingly, we observed strong allele-specific expression differences of both of these genes at SNP loci in the coding-regions, rs3761764 (*p*-value = 0.031) and rs2061242 (*p*-value = 1.35E-07), respectively (Figure 4B).

These results suggest that the rs151202 variant at chr5:54529604 likely disturbed *TFAP2C* binding, resulting in differential expression of *DHX29* and *SKIV2L2* genes. Interestingly, *TFAP2C* is a key regulator of hormone responsiveness in breast carcinoma cells through the control of multiple estrogen signaling pathways (Gee et al., 2009). It is also noteworthy that the overexpression of *DHX29* can promote cancer cell growth in culture and in xenografts (Parsyan et al., 2009). Taken together, these results demonstrate that regSNPs-ASB has the potential to identify causal regulatory SNPs that affect TF binding and further impact the expression levels of target genes.

### Correlation of Allele-Specific TF Binding and Allele-Specific Gene Expression

We next asked whether the regulatory SNPs identified by regSNPs-ASB could be found in a list of eQTL signals. To address this question, we compared all of the regulatory SNPs identified in MCF-7 and MSC to the eQTLs in the GTEx database. We found that 30.2% (16/53) of the regulatory SNPs in MCF-7 and 42.6% (52/122) of the regulatory SNPs in MSC were also eQTLs (Supplementary Tables S3, S4). This enrichment was significant when compared to non-regulatory SNPs (chi-square test, *p*-value = 1.52E-08). Thus, SNPs identified by regSNPs-ASB that are also in eQTLs could be prioritized for experimental validation of their functional role as the causal variants involved in the target gene regulation.

**FIGURE 5:**
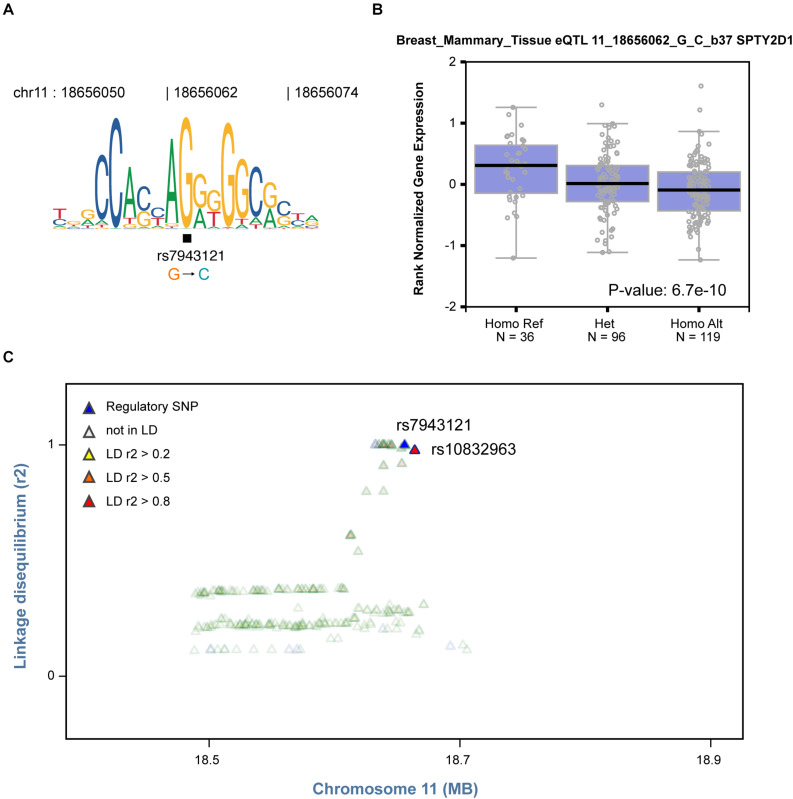
Identified regulatory SNP is in strong LD with a GWAS SNP in breast cancer. **(A)** The C allele of regulatory SNP rs7943121 is predicted to compromise binding of the CTCF TF based on the motif, which is consistent with regSNP-ASB predictions. **(B)** The genotype of regulatory SNP rs7943121 is correlated with SPTY2D1 gene expression (GTEx breast cancer data). Each dot represents the expression of a sample. The sample groups of different rs7943121 genotype were indicated on the *X*-axis; and the relative expression level of SPTY2D1 is shown on the *Y*-axis. The median value of SPTY2D1 expression level in each genotype group is represented by the dark black horizontal line in the box plot. Homo Ref: homozygote for the reference G allele, Het: heterozygote; Homo Alt: homozygote for the alternative allele. A chi square *P*-value was calculated based on Mahalanobis distance. **(C)** The regulatory SNP rs7943121 is in strong LD (*r*^2^ > 0.8) with SNP rs10832963 (red).

Based on the finding that regulatory SNPs are significantly enriched in eQTLs, we further tested whether our method could be used to identify plausible causal SNPs that modulate TF binding from a list of GWAS SNPs associated with specific cell systems. Notably, rs7943121 was identified as a SNP that weakens the binding of the transcriptional repressor CCCTC-binding factor (CTCF) (FDR = 0.033, Figure 5A). This SNP was also identified as an eQTL in breast mammary tissue that could modulate *SPT2 chromatin protein domain containing 1* (*SPTY2D1*) expression (Figure 5B). Furthermore, we also observed that rs7943121 was in strong LD (*r*^2^ > 0.8) with rs10832963, which is one of the most significant GWAS-SNPs associated with breast cancer (Figure 5C; Michailidou et al., 2017). Overall, these results provide compelling evidence that regulatory SNPs detected by regSNPs-ASB can potentially be the drivers of previous-reported eQTL and GWAS signals.

## Discussion

eQTLs and GWAS are conventional methods for mapping association variants that have identified tens of thousands of loci that are highly correlated with gene expression and common diseases, respectively. However, screening for the true causal variants remains a challenging problem. In this study, we introduced a computational method, regSNPs-ASB, for detecting regulatory SNPs that affect transcription factor binding using ATAC-seq data. The major conclusion of this study is that by integrating ATAC-seq data with RNA-seq expression data, chromatin conformation data and LD information from SNP datasets, regSNPs-ASB is able to efficiently evaluate the regulatory functions of SNPs in disease contexts. This conclusion is based on the following evidence: First, we systematically detected potential regulatory SNPs with allele-specific differences in TF occupancy using a statistical test. Second, we provided evidence that regulatory SNPs can affect the expression of their target genes. Finally, we found that regulatory SNPs are significantly enriched within expression-correlated variants compared with non-regulatory SNPs in the GTEx database. Collectively, these findings demonstrate that the regSNPs-ASB algorithm can be used to identify causal variants from ATAC-seq data, and thereby, further our understanding of the molecular mechanisms of complex diseases.

Our method is developed based on the established strategy for conducting allele-specific analysis on many types of sequencing data, which specifically focus on the sequencing reads on heterozygous loci. This includes allele-specific gene expression (Zhang et al., 2014; Castel et al., 2015), allele-specific alternative splicing analysis (Nembaware et al., 2008; Li et al., 2012), allele-specific binding of ChIP-seq (de Santiago et al., 2017) and CLIP-seq data analysis (Yang et al., 2019), allele-specific chromatin interaction (Cavalli et al., 2019), and allele-specific chromatin accessibility (Harvey et al., 2014; Zhang et al., 2019). To our knowledge, our method is the first to analyze allele-specific footprint.

When designing regSNPs-ASB, we considered several potential issues that could arise from technical artifacts. On the one hand, the intrinsic sequence bias of DNase I and Tn5 enzymes has a striking effect on the average cut profile over a specific TF-binding motif, which is a major limitation of current genomic footprinting methods (Yardımcı et al., 2014; Xu et al., 2017). regSNPs-ASB effectively eliminates the impact of sequence preference bias, since this algorithm focuses on identifying the differences in the cut-site distribution between two alleles of a single locus, where the technical variability should be the same. For the same reason, our model is insensitive to the differences in the intrinsic footprint patterns from different TF domains. On the other hand, cancer cell lines often contain copy-number variants, which complicate the detection of allele-specific TF binding. Traditional ChIP-based methods for identifying allele-specific TF binding (Wei et al., 2012; Cavalli et al., 2016), cannot effectively distinguish gene expression changes that are caused by allele-specific copy number amplification from the effects caused by allele-specific TF binding. regSNPs-ASB ignores this feature of cancer genomes because it detects differences in footprint shape that result from TF-binding, rather than the imbalance of read counts from different alleles. By avoiding these technical problems, regSNPs-ASB effectively screens for allele-specific TF binding in an unbiased manner.

We also note that there are some limitations of regSNPs-ASB. First, although this model can effectively evaluate TFs with shallow footprints that are caused by transient interactions with DNA, regSNPs-ASB, like all other footprinting-based computational algorithms, cannot detect TFs that do not leave footprints because of their short occupancy time (on the order of seconds). In that case, the differential TF binding events cannot be detected by ATAC-seq and technological improvements will be needed to capture those events. Second, the proposed method can only be applied on the heterozygous loci in the genome, and cannot be used to identify the functional variants on the homozygous variants. In addition, regSNPs-ASB infers TF occupancy from open chromatin regions that contain a probabilistic match to TF consensus recognition motifs, which limits the ability to identify TFs whose motif sequences are not known. However, this limitation will diminish as new TF motifs are discovered and included in the JASPAR database.

It should be noted that the proposed methods are designed for the cell systems with diploid genomes, which may not be appropriate to study the cell systems with complicated chromosome rearrangement, including tumors and cancer cells that have aberrant CNV (copy number variation) and LOH (lost of heterogeneity). In theory, our method can be applied on the genomic loci with somatic mutations. At such loci, however, since only a proportion of the cells will carry the same mutation, the power of our method for detecting such variants may be compromised.

In summary, we show that the regSNPs-ASB algorithm is effective in identifying candidate causal SNPs from ATAC-seq data. This new computational tool will enable efficient prioritization of genetic variants identified by association studies for further studies to validate their causal regulatory function. Ultimately, identifying causal genetic variants will further our understanding of the underlying molecular mechanisms of disease.

## Materials and Methods

### Cell Lines

Human MCF-7 breast cancer cells were purchased from ATCC (Manassas, VA, United States) and were authenticated using cell line authentication services of Genetica (Burlington, NC, United States). Cells were maintained in minimal essential media (MEM) plus 10% fetal bovine serum (FBS) with penicillin and streptomycin. Media was changed to phenol red-free MEM with 5% charcoal-dextran treated FBS for at least 3 days prior to experiments. We generated 3 ATAC-seq libraries and 2 RNA-seq libraries from MCF-7 cells. Each individual library was derived from three technical replicates. Human mesenchymal stem cells (MSCs) were purchased from Lonza (Walkersville, MD, United States) and were tested for purity by flow cytometry. MSCs were thawed and the culture process was initiated by plating in tissue culture flasks (Corning, Corning, NY, United States) containing MSC growth medium (Lonza) at 37°C in 5% CO_2_-90% humidity according to the manufacturer’s instructions. We generated 3 ATAC-seq libraries from MSC and each individual library was derived from two technical replicates.

### ATAC-seq Experimental Procedure

Assay for transposase-accessible chromatin with high-throughput sequencing was performed according to the published protocol (Buenrostro et al., 2013). Briefly, cells were collected in cold PBS and cell membranes were disrupted in cold lysis buffer (10 mM Tris–HCl, pH 7.4, 10 mM NaCl, 3 mM MgCl_2_ and 0.1% IGEPAL CA-630). The nuclei were pelleted and resuspended in the transposase reaction mix containing 25 μL 2 × TD buffer, 2.5 μL transposase (Illumina) and 22.5 μL nuclease-free water. Directly following transposition, the sample was purified using a Qiagen MinElute kit. Following purification, libraries were amplified using 1 × NEBnext PCR master mix and 1.25 μM custom Nextera PCR primers. AMPure XP beads (Beckman Coulter) were used to purify the transposed DNA and the amplified PCR products. All libraries were sequenced on a 100-cycle paired-end run on an Illumina NovaSeq 6000 instrument.

### RNA-seq Experimental Procedure

Total RNA was prepared using a RNeasy kit (Qiagen). The concentration and quality of total RNA samples was first assessed using an Agilent 2100 Bioanalyzer. A RIN (RNA integrity number) of five or higher was required to pass the quality control. A TruSeq Stranded mRNA Library Prep Kit (Illumina) was used to prepared single-indexed strand-specific cDNA libraries from 500 nanograms of RNA per sample. The resulting libraries were quantified using a Qubit and the size distribution was assessed using an Agilent 2100 Bioanalyzer. Pooled libraries (1.5 picomoles) were sequenced with 2 × 75 bp paired-end configuration on a HiSeq 4000 instrument (Illumina). A Phred quality score (Q score) was used to measure the quality of sequencing. More than 90% of the sequencing reads reached Q30 (99.9% base call accuracy).

### Preprocessing for Identifying Allele-Specific Transcription Factor Binding

For ATAC-seq footprinting analysis, all of the read start sites were adjusted to represent the center of the transposon binding event; reads aligning to the forward strand were offset by + 4 bp and reads aligning to the reverse strand were offset by -5 bp (Buenrostro et al., 2013). MACS2 (Feng et al., 2011) was used with default parameters to identify all ATAC-seq peaks. Peaks with <200 cutting sites were removed from downstream analysis to minimize the effect of nucleosome-bound regions. Heterozygous SNPs were identified using samtools-1.6 and bcftools-1.6 with the parameters *mpileup -uf* and *view -Nvcg*, respectively (Li et al., 2009). SNPs were further filtered by VcfFilter with the parameter *DP* > *10* and *MQ* > *20* (Erik, 2012). In each open chromatin region, we used FIMO (Grant et al., 2011) to recognize potential TF binding sites from both reference and alternative alleles. Lastly, we used BEDTools *IntersectBed* (Quinlan and Hall, 2010) to merge the loci of heterozygous variants and potential TF binding sites. The set of TF binding sites intersecting SNPs were regarded as potential allele-specific TF binding sites.

### Transcription Factor Motif Disruption Analysis

The position-specific scoring matrices (PSSM) of the candidate TFs were retrieved from the JASPAR database (Khan et al., 2018) and used to annotate the potential regulatory effects of the tested SNPs on TF motifs. The magnitude of the change in binding affinity was calculated as the absolute difference (delta) of PSSM scores, that is delta (PSSM) = PSSM(ref) – PSSM(alt).

### regSNPs-ASB Computational Model Description

We explicitly modeled the counts of DNA fragments for each potential binding site using a generalized linear model. The total number of sequencing reads within a given region of the genome approximately follow a negative binomial distribution.

For each potential allele-specific TF binding site, we built the following generalized linear regression model to fit the DNA fragment counts:

$$\log\left(E\left(y\right)\right)=\beta_{0}+\beta_{r}x_{r}+\beta_{a}x_{a}+\beta_{\mathrm{int}}x_{r}x_{a}+\varepsilon$$

where y is the number of ATAC-seq reads in which the Tn5 cleavage position mapped to a specific region and allele; x_r_ and x_a_ are binary predictor variables that indicate the region where a cleavage event happened (0 = binding site and 1 = flanking region) and the allele that the fragment mapped to (0 = reference allele and 1 = alternative allele), respectively (Figure 1B); β_r_ and β_a_ are the regression coefficients used to estimate the relationship between the scalar response and x_r_ and x_a_. In addition, β_0_ indicates the average sequencing depth around a heterozygous site and ε is the random error. Logarithm is the canonical link function when the response variable follows a negative binomial distribution. Our null hypothesis (H_0_) is: β_int_ = 0. Rejecting the null hypothesis indicates that the allelic imbalance differs between reference and alternative alleles. A positive or negative β_int_ value indicates a gain or loss of binding ability to the variant, respectively. False discovery rate was calculated using the Benjamini-Hochberg procedure (Benjamini and Hochberg, 1995).

### Regulatory SNPs Target Gene Identification

For each regulatory SNP, we first used SnpSift (Cingolani et al., 2012) to retrieve the corresponding reference SNP ID number (rsID) before querying the SNPs to the Short Genetic Variations database (dbSNP). ANNOVAR (Hakonarson et al., 2010) was used to annotate the location of each SNP. Variants located within a 1 kb region upstream or downstream of a transcription start site were considered as potential promoter regions. The remaining regulatory SNPs were considered as enhancers using chromatin interaction data from the 4DGenome database^2^. Each record in this database includes three parts: a chromatin interaction between two genomic regions, genes located in the interacting regions, and the experimentally-derived or computationally-predicted data used to detect the genomic interaction. BEDTools *IntersectBed* (Quinlan and Hall, 2010) was used to examine overlaps between regulatory SNPs and interacting chromatin regions. For any genomic region overlapping with regulatory SNPs, genes located in the paired interacting region were recognized as potential target genes for the corresponding regulatory SNPs.

### Allele-Specific Expression Analysis

Typical allele-specific expression analysis seeks to capture allelic imbalance of reference and alternative alleles in RNA-seq read counts covering heterozygous sites. Under the null hypothesis of balanced expression, the fraction of allelic read counts is expected to fit a binomial distribution (N, 0.5). For each regulatory SNP target gene, we used a binomial test to detect whether allele-specific expression occurred at each coding SNP based on the corresponding RNA-seq data. All target genes that reject the null hypothesis (*P* value < 0.05) were considered genes with allele-specific expression.

### SNP Linkage Disequilibrium Analysis

We used the 1000 Genome Project Phase 3 variants^3^ (Auton et al., 2015) and plink2 (Chang et al., 2015) to conduct the linkage disequilibrium analysis. First, vcf file formats were converted to the corresponding pgen, psam, and pvar file formats using *plink –vcf*. Then we calculated *r*^2^ and D’ between each SNP and disease-correlated GWAS-SNP using *plink –out*. The linkage disequilibrium results were plotted by the genetic variant-centered annotation browser, SNiPA (Arnold et al., 2015).

### eQTL Analysis

The eQTL analysis was performed using GTEx v7 data^4^. We classified a regulatory SNP as a genetic variant exerting regulatory effects on the expression of gene if it was completely overlapping with any eQTL in the corresponding tissue.

## Data Availability Statement

The datasets presented in this study can be found in online repositories. The names of the repository/repositories and accession number(s) can be found in the article/Supplementary Material.

## Author Contributions

YL and SX conceptualized the project and drafted the manuscript. SX performed the bioinformatic analyses. SX, JR, ZL, CY, and YL wrote and edited the manuscript. HN, HG, YW, and YL contributed to the design of the experiments and data interpretation. WS participated in the development of methodology. YL and WF gave the research direction. All authors contributed to the article and approved the submitted version.

## Conflict of Interest

The authors declare that the research was conducted in the absence of any commercial or financial relationships that could be construed as a potential conflict of interest.

## Footnotes

1 https://yunlongliulab.shinyapps.io/regsnps-asb/

2 https://4dgenome.research.chop.edu/

3 hgdownload.cse.ucsc.edu/gbdb/hg19/1000Genomes/phase3/

4 https://gtexportal.org/home/datasets

## Supplementary Material

FIGURE S1 **(A)** The histogram of the number of heterogeneous variants on each TF in MSC. **(B)** The histogram of the number of TF binding sites on each variant in MSC. **(C)** The histogram of the number of heterogeneous variants on each TF in MCF7. **(D)** The histogram of the number of TF binding sites on each variant in MCF7.

TABLE S1 GLM results of MCF-7.

TABLE S2 GLM results of MSC.

TABLE S3 eQTL analysis and regulatory SNP annotation of MCF-7.

TABLE S4 eQTL analysis and regulatory SNP annotation of MSC.

TABLE S5 Raw RNA-seq data of MCF-7.

TABLE S6 Chromatin interaction experimental data of MCF-7.

TABLE S7 ASE analysis for the target genes of regulatory SNPs in MCF-7.
