# Supplementary figures and images for: regSNPs-ASB: A Computational Framework for Identifying Allele-Specific Transcription Factor Binding From ATAC-seq Data
Source: Front Bioeng Biotechnol. 2020 Jul 29;8:886. doi: 10.3389/fbioe.2020.00886 (PMC7405637; doi:10.3389/fbioe.2020.00886)

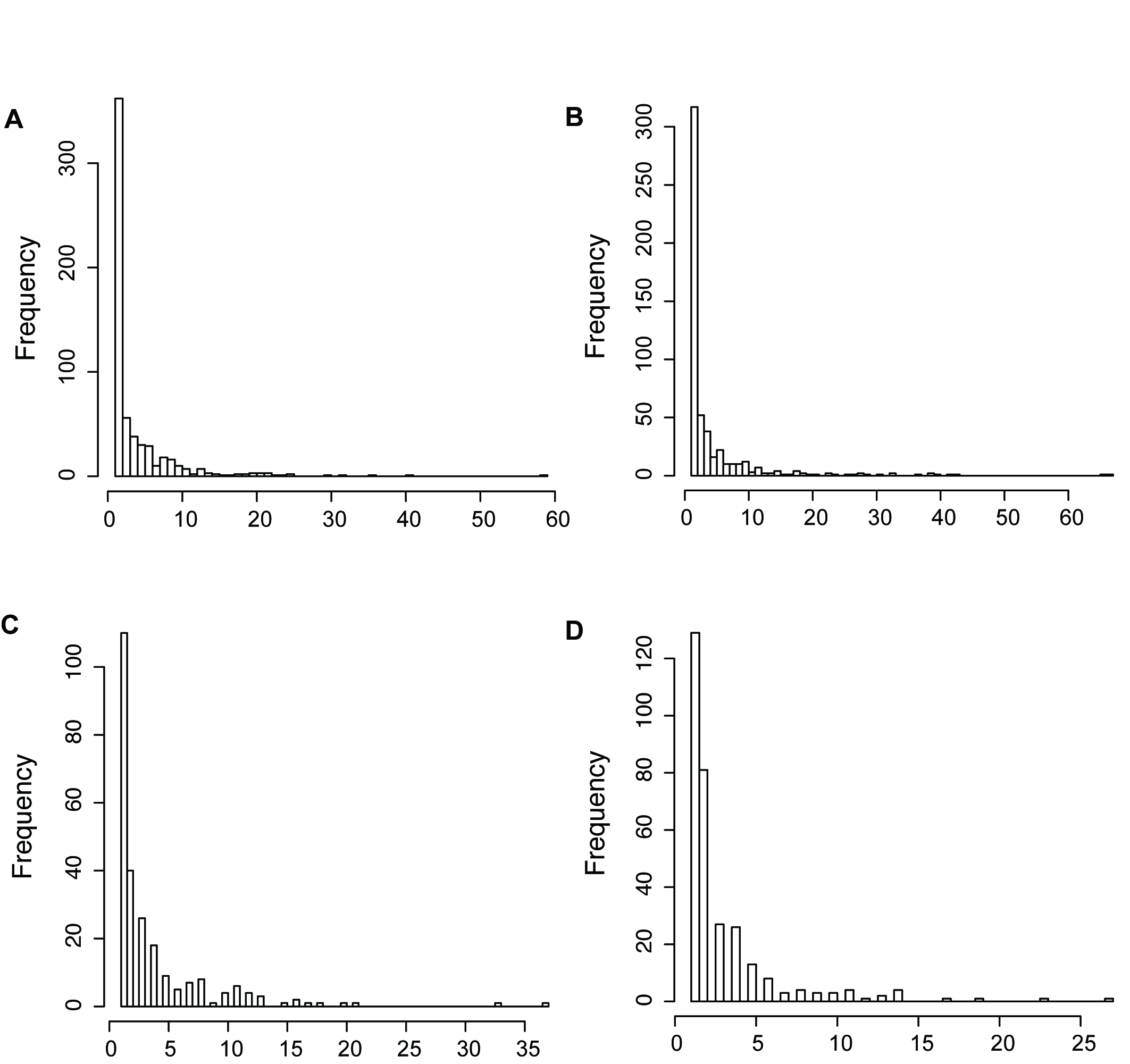

Supplement: FIGURE S1 — (A) The histogram of the number of heterogeneous variants on each TF in MSC. (B) The histogram of the number of TF binding sites on each variant in MSC. (C) The histogram of the number of heterogeneous variants on each TF in MCF7. (D) The histogram of the number of TF binding sites on each variant in MCF7. [file Image_1.TIF]
